# Treatment with a DNA methyltransferase inhibitor feminizes zebrafish and induces long-term expression changes in the gonads

**DOI:** 10.1186/s13072-017-0168-7

**Published:** 2017-12-08

**Authors:** Laia Ribas, Konstantinos Vanezis, Marco Antonio Imués, Francesc Piferrer

**Affiliations:** 1 Institut de Ciències del Mar, Consejo Superior de Investigaciones Científicas (CSIC), Passeig Marítim, 37–45, 08003 Barcelona, Spain; 2 Imperial Centre for Translational and Experimental Medicine, Hammersmith Hospital, Du Cane Road, London, W12 0NN UK; 3 Departamento de Recursos Hidrobiológicos, Universidad de Nariño, Torobajo, Pasto, Colombia

**Keywords:** 5-Aza-dC, Methylation, dnmt, Sex ratio, Epigenetic, Reproduction, Zebrafish

## Abstract

**Background:**

The role of epigenetic modifications such as DNA methylation during vertebrate sexual development is far from being clear. Using the zebrafish model, we tested the effects of one of the most common DNA methyltransferase (dnmt) inhibitor, 5-aza-2′-deoxycytidine (5-aza-dC), which is approved for the treatment of acute myeloid leukaemia and is under active investigation for the treatment of solid tumours. Several dose–response experiments were carried out during two periods, including not only the very first days of development (0–6 days post-fertilization, dpf), as done in previous studies, but also, and as a novelty, the period of gonadal development (10–30 dpf).

**Results:**

Early treatment with 5-aza-dC altered embryonic development, delayed hatching and increased teratology and mortality, as expected. The most striking result, however, was an increase in the number of females, suggesting that alterations induced by 5-aza-dC treatment can affect sexual development as well. Results were confirmed when treatment coincided with gonadal development. In addition, we also found that the adult gonadal transcriptome of 5-aza-dC-exposed females included significant changes in the expression of key reproduction-related genes (e.g. *cyp11a1*, *esr2b* and *figla*), and that several pro-female-related pathways such as the Fanconi anaemia or the Wnt signalling pathways were downregulated. Furthermore, an overall inhibition of genes implicated in epigenetic regulatory mechanisms (e.g. *dnmt1*, *dicer*, *cbx4*) was also observed.

**Conclusions:**

Taken together, our results indicate that treatment with a DNA methylation inhibitor can also alter the sexual development in zebrafish, with permanent alterations of the adult gonadal transcriptome, at least in females. Our results show the importance of DNA methylation for proper control of sexual development, open new avenues for the potential control of sex ratios in fish (aquaculture, population control) and call attention to possibly hidden long-term effects of dnmt therapy when used, for example, in the treatment of prepuberal children affected by some types of cancer.

**Electronic supplementary material:**

The online version of this article (10.1186/s13072-017-0168-7) contains supplementary material, which is available to authorized users.

## Background

DNA methylation is one of the main epigenetic modifications involved in gene expression regulation. In vertebrates, it consists in the addition of a methyl group to the 5′ position of cytosine followed by a guanine (CpG) [1, 2]. Proper control of DNA methylation is essential for many phenomena, including X-chromosome inactivation [3], genomic imprinting [4] or ageing [5]. DNA methylation is carried out by enzymes named DNA methyltransferases (dnmts) [6]. In mammals, the main dnmts include one responsible for DNA methylation maintenance (*dnmt1*) and another two for de novo DNA methylation (*dnmt3a/b*) [7, 8]. Addition of methyl groups to CpGs by dnmts can prevent transcription factor binding and hence gene expression [6, 9]. Many studies have focused on the consequences of DNA methylation alterations by using dnmt inhibitor agents to control the expression of genes involved in the onset of cancer [10]. Furthermore, many tumour cells have hypermethylation in the promoters of tumour suppressor genes [11, 12] and thus research has also contemplated the effects of demethylating agents to regain the expression of these silenced genes [13, 14].

The most popular demethylation agents are 5-azacytidine (5-aza-CR), 5-aza-2′-deoxycytidine (5-aza-dC), commonly named as decitabine, and zebularine. 5-Aza-dC is more potent than 5-aza-CR, but both are more toxic and unstable than zebularine [14, 15]. These agents block DNA methylation when incorporated in the DNA as cytidine nucleoside analogues [16], forming a covalent bond in which dnmts become removed from the active nuclear pool and the genome results hypomethylated [17, 18]. However, despite many studies on the underlying biochemical reactions taking place in cells exposed to these agents, their exact in vivo mechanism still remains unclear [18, 19].

In recent years, zebrafish (*Danio rerio*) has become widely accepted as a model for the study of epigenetic regulatory mechanisms, which are generally conserved with respect to those of mammals [20, 21]. Thus, for example, epigenetic alterations that occur during germ cell development are common between mice and zebrafish [22, 23]. The paternal zebrafish methylome is inherited through the sperm. After fertilization, the maternal zebrafish methylome is reprogrammed to match the paternal methylome [22, 24]. Subsequently, during earlier development stages, about 80% of the CpGs in the zebrafish genome are methylated with some fluctuations along development, i.e. blastula and gastrula stages [25, 26].

Few studies have investigated the effects of dnmt inhibitors in fish models. In zebrafish, demethylation agents have been used to better understand the role of DNA methylation during early development, where lack of proper DNA methylation resulted in different types of malformations [27]. Cranial deformities were also observed in another fish model, the Japanese rice fish (*Oryzias latipes*), after early exposure to 5-aza-CR [28]. As it occurs in mammalian cells [17, 29], treatment of zebrafish with 5-aza-CR results in global hypomethylation in embryonic cells [30] as well as in adult hepatocytes [31]. In female zebrafish fed by 5-aza-dC during 32 days a decrease in global DNA methylation was observed [32], likewise, in larvae treated with 10 or 25 µM during 0–6 dpf [33]. However, transgenerational effects up to the F2 generation were only observed in the latter study [33]. Few data are found in parental imprinting in fish gametes (reviewed in [34]), a process responsible for the heritance of the DNA methylome. In zebrafish, it has been documented that dynamic changes in DNA methylation occur during imprinting [25] and that the DNA methylome is inherited through the sperm, but no through the oocyte [22].

During the last few years, the importance of epigenetic regulatory mechanisms for sexual development has been realized, particularly in organisms where sex is the result of the interplay between genetic and environment (reviewed in [35]). Thus, in fish the methylation levels of the promoter of gonadal aromatase (*cyp19a1a*)—the enzyme that converts androgens to oestrogens—in the European sea bass, *Dicentrarchus labrax*, were positively correlated with temperature during early development [36]. In the olive flounder, *Paralichthys olivaceus* [37] and in zebrafish [38] *cyp19a1a* methylation levels during ovarian development have been studied, showing different methylation patterns during folliculogenesis. Whole-genome approaches have revealed global hypermethylation in various chromosomes in the gonads of Nile tilapia, *Oreochromis niloticus*, exposed to elevated temperatures when compared to control fish [39]. Also, in the half-smooth tongue sole, *Cynoglossus semilaevis*, genome-wide DNA methylation analysis revealed the existence of an epigenetic regulatory mechanism on the suppression of the female-specific W chromosomal genes in high-temperature masculinized fish [40]. However, the role of DNA methylation during gonadal development is far from being clear.

The zebrafish is also increasingly becoming a useful model for aquaculture-related research, where, for example, the control of sex ratios is pursued due to the frequent sexual dimorphism in growth [41]. Domesticated zebrafish have a polygenetic sex-determining system in which genetic factors in combination with environmental factors determine the sexual phenotype [42, 43]. In contrast, wild zebrafish has a chromosomal (WZ/ZZ) sex determination system [44]. Thus, domesticated zebrafish is a well-suited model for studying the effects of environmental perturbations on its development, particularly sexual development. After preliminary trials, in this study we report the establishment of the appropriate conditions for treatment of zebrafish with the most common dnmt inhibitor, 5-aza-dC. Importantly, treatments were not limited to embryonic development, as done earlier, but included treatments covering the period of gonadal development. We report the effects of dose, timing and duration of treatment with 5-aza-dC in terms of resulting survival, deformities and growth. Interestingly, we show that treatment with 5-aza-dC consistently results in an increase in the number of females after different treatments, opening the possibility for a new approach to study the epigenetic regulation of sex and its control, and provide a detailed description of the effects on the gonadal transcriptome as a result of 5-aza-dC treatment. We also raise the possibility that some of the novel effects found in zebrafish ovaries could also be happening in other vertebrates, including humans, particularly prepuberal children affected by some types of cancer, where treatment with DNA-demethylating agents is clinically used.

## Results

### 5-Aza-dC decreases survival and induces teratologies when administered during zebrafish early development

#### Early development experiments

Treatment of zebrafish eggs with 5-aza-dC at 0, 5, 15 or 25 µM added to the embryo medium from 0 to 6 days post-fertilization (dpf) resulted in a progressive decrease in survival at the end of the treatment: 75.3, 62, 66 and 44%, respectively. At 30 dpf, these survival values had further dropped to 34, 42, 44 and 26%, respectively.

**Fig. 1:**
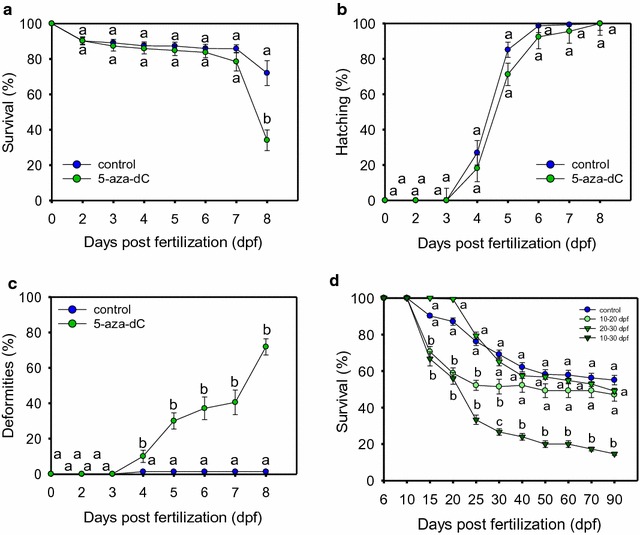
Zebrafish treated with 5-aza-dC. **a** Survival, **b** hatching rate and **c** teratology of zebrafish embryos treated with 5-aza-dC at 75 µM from 0 to 2 dpf. Each shown datapoint is the mean ± s.e.m. of seven independent experiments. Within each experiment, each datapoint is the mean of three technical replicates. Significant differences *P* < 0.05 (in **a**) or *P* < 0.01 (in **c**) among groups at a given age are indicated by different letters and were examined by Student’s *t* test. **d** Survival of treated zebrafish with 25 µM of 5-aza-dC during gonadal development. Each datapoint is the mean ± s.e.m. of two independent experiments. Within each experiment, each datapoint is the mean of 2–4 technical replicates, originated from five breeding pairs. Significant differences (*P* < 0.05) among groups at a given sampling age were tested by one-way ANOVA and are indicated by different letters

**Fig. 2:**
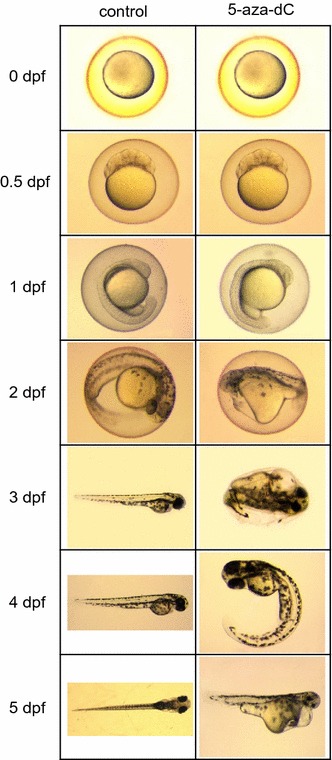
Representative teratology observed from zebrafish embryos treated with 5-aza-dC at 75 µM from 0 to 2 dpf. Teratology was observed from 2 dpf in two out of seven families, and between 3 and 4 dpf, it was observed in all tested families. Different types of teratologies were observed; body curvature, problems with yolk-sac reabsorption and body deformation

Treatment of eggs with 5-aza-dC at 75 µM from 0 to 2 dpf resulted in significantly (*P* < 0.05) lower survival at 8 dpf but not before (Fig. 1a). A non-significant delay on development, as assessed by hatching rate, was also observed (Fig. 1b). Teratologies were already observed at 2 dpf in two out of seven tested families, but in all of them teratology was observed between 3 and 4 dpf onwards (Fig. 2); however, significant differences (*P* < 0.05) were not found until 4 days and onwards, with ~ 75% of the surviving treated fish affected at 8 dpf (*P* < 0.05; Fig. 1c). Teratologies included three major types: body curvature, reduced yolk-sac reabsorption and overall body deformation (Fig. 2).

#### Gonadal development experiments

Treatment of larvae with 5-aza-dC at 25 µM at different periods during gonadal development resulted in significantly (*P* < 0.05) lower survival, particularly if the treatment started at 10 dpf and lasted until 30 dpf (Fig. 1d). Differences in survival persisted until the end of the experiment at 90 dpf only in the fish treated from 10 to 30 dpf. Survival of fish treated only between 10 and 20 dpf was also significantly (*P* < 0.05) reduced from 15 to 30 dpf but stabilized at 40 dpf, without differences with the controls. No differences in fish survival were found in the 20–30-dpf period when compared to controls (Fig. 1d).

The survival of fish treated with 5-aza-dC at the highest concentration (75 µM) from 10 to 30 dpf was reduced to 56% one week after starting the treatment (17 dpf) and to 42.6% at the end of the treatment (30 dpf). At 90 dpf, survival of treated fish was only 13.3%. Furthermore, surviving fish were smaller than the controls (Additional file 1: Fig. S1A) both in standard length (SL) in males (*P* < 0.05) and in females (*P* < 0.01) and in body weight (BW) in males (*P* < 0.05) and females (*P* = 0.08) (Additional file 1: Fig. S1 B, C).

### 5-Aza-dC treatment consistently alters the sex ratio

**Fig. 3:**
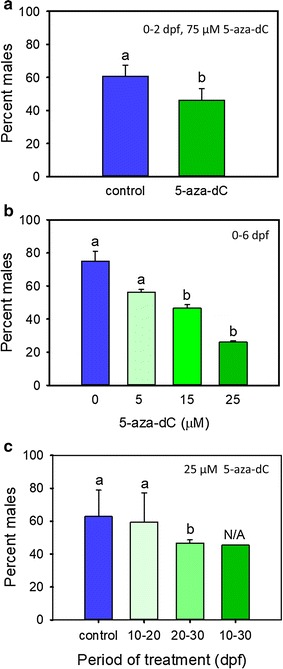
Effects of treatment with 5-aza-dC on the resulting sex ratio at 90 dpf. **a** Sex ratio of fish treated with 5-aza-dC at 75 µM from 0 to 2 dpf. Each datapoint is the mean ± s.e.m., corresponding to five independent experiments using five different breeding pairs. Numbers of fish are 162 and 81 in control and 5-aza-dC, respectively. Significant differences among groups (*P* < 0.05) were analysed by the Chi-squared test with Yate’s correction. **b** Sex ratio of zebrafish larvae (0–6 dpf) treated with different 5-aza-dC doses (0, 5, 15, 25 µM *n* = 13, 27, 31, 19, respectively). Each datapoint is the mean ± s.e.m. of two independent experiments with two technical replicates each. **c** Sex ratio of zebrafish treated with 25 µM of 5-aza-dC during different periods during gonadal development. Data shown as mean ± s.e.m. of three independent experiments with 1–3 biological replicates (*n* = 133, 59, 72 and 11 in control, 10–20 dpf, 20–30 dpf and 10–30 dpf groups, respectively). Significant differences among groups were analysed by the Chi-squared test with Yate’s correction. Significant differences (*P* < 0.05) are indicated by different letters. N/A, variation not assessed because there was only one experiment one technical replicate

In this study, the number of males in the control groups of the different experiments was in the range of 60–75%, a typical value for domestic zebrafish (AB strain). Treatment with 5-aza-dC at 75 µM from 0 to 2 dpf significantly (*P* < 0.05) reduced the number of males at 90 dpf (Fig. 3a). Furthermore, a clear dose–response effect was elicited when treatment was carried out from 0 to 6 dpf (Fig. 3b), with significant differences (*P* < 0.05) in the number of males with respect to the untreated controls observed with the 15- and 25-µM doses. Consistent with these results, the number of males also decreased in fish treated with 5-aza-dC at 25 µM when the treatment included the 20–30- or 10–30-dpf periods; however, significant differences (*P* < 0.05) could be recorded only in the 20–30 dpf due to the lack of replication in the 10–30-dpf period. In contrast, no significant differences in sex ratio were observed when treatment took place during the 10–20-dpf period. As stated in the previous section, larvae treated with 5-aza-dC fish at 75 µM from 10 to 30 dpf had very low survival. Therefore, in this group sex ratios could not be assessed accurately due to the low number of fish available for statistics. Taken together, the data shown above indicate that treatment with 5-aza-dC is able to alter sexual development in zebrafish.

### Long-term effects of 5-aza-dC treatment on the expression of dnmt1 and dnmt3b

**Fig. 4:**
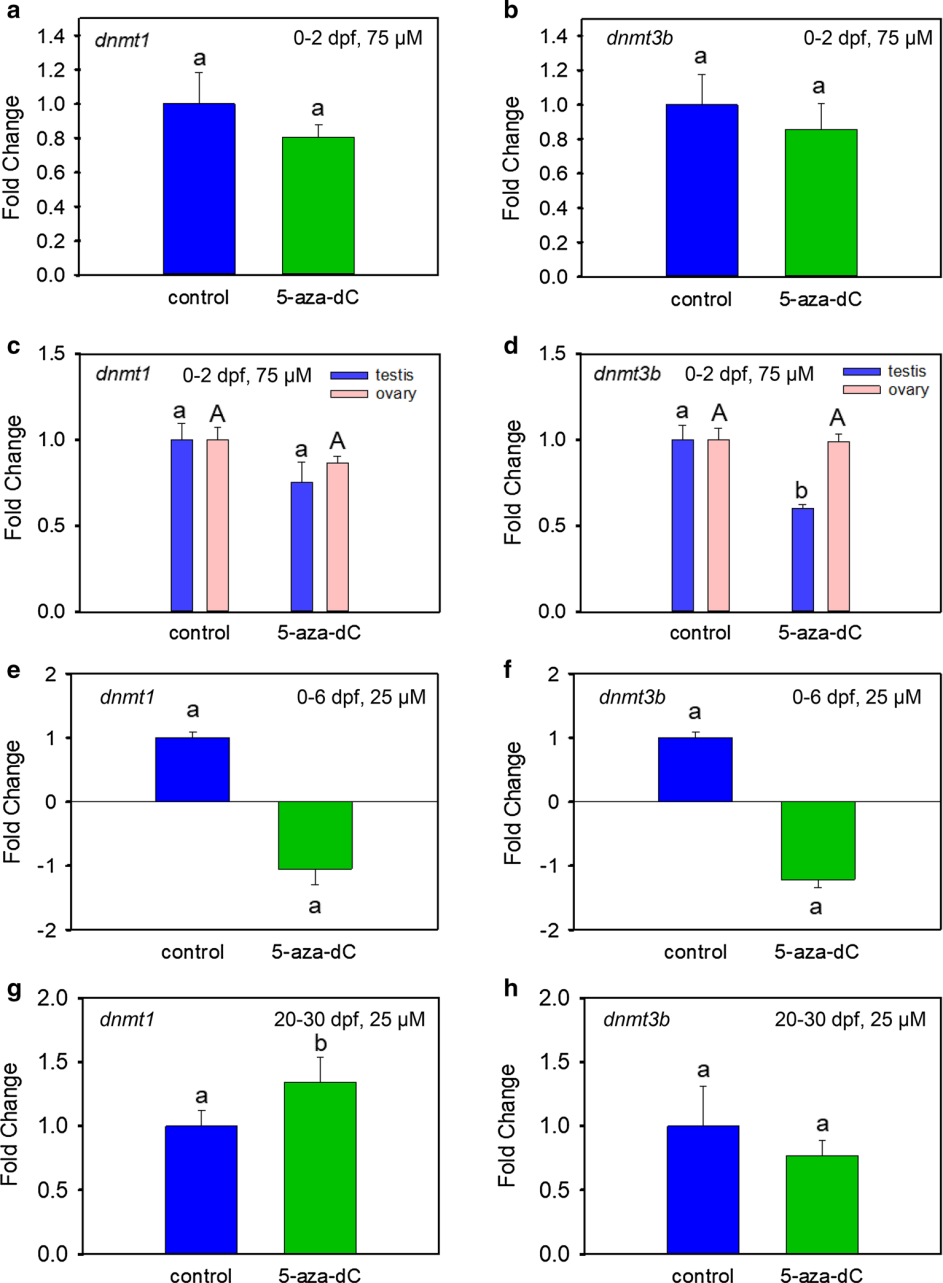
Gene expression profiles of DNA methyltransferases 1 and 3 (*dnmt1* and *dnmt3b*) in zebrafish treated with 5-aza-dC. **a**, **b** 0–2-dpf treatment, short-term effects. Gene expression of *dnmt1* (**a**) and *dnmt3b* (**b**) in larvae at 4 dpf previously treated with 5-aza-dC at 75 µM from 0 to 2 dpf. Each datapoint is the mean ± s.e.m., corresponding to 5 pools of larvae each, in turn, made of a pool of ~ 20 larvae from two independent experiments. **b**, **c** 0–2-dpf treatment, long-term effects. Gene expression of *dnmt1* (**c**) and *dnmt3b* (**d**) in zebrafish gonads at 90 dpf after treatment with 5-aza-dC at 75 µM from 0 to 2 dpf. Data shown as mean ± s.e.m. of fold change using control values set at 1. Sample size *n* = 7–9 gonads per sex and treatment. Within the same sex, different letters indicate significant differences (*P* < 0.01) between treated and control fish analysed by Student’s *t* test. **e**, **f** 0–6-dpf treatment, mid-term effects. Gene expression of *dnmt1* (**e**) and *dnmt3b* (**f**) in juvenile fish at 30 dpf treated with 5-aza-dC at 25 µM in the period 0–6 dpf. Each datapoint is the mean ± s.e.m. with *n* = 7 individual larvae corresponding to 3 technical replicates. The same letter between groups indicates no significant differences (*P* > 0.05) among groups were tested by Student’s *t* test. **g**, **h** 20–30-dpf treatment, short-term effect. Gene expression of *dnmt1* **g** and *dnmt3b* **h** in body trunks of juvenile zebrafish at 30 dpf after treatment from 20 to 30 dpf with 25 µM 5-aza-dC. Data shown as mean ± s.e.m. fold change of *n* = 12 samples per group using control values set at 1. Significant differences (*P* < 0.05) are indicated by different letters

Treatment at 75 µM from 0 to 2 dpf did not affect the expression of the *dnmt1* (Fig. 4a) and *dnmt3* (specifically, *dnmt3bb.2*, which will be referred to as *dnmt3b* in the rest of the paper for simplicity) (Fig. 4b) in 4-dpf larvae. In addition, 5-aza-dC did not alter the expression of *dnmt1* in the gonads of 90-dpf adults (Fig. 4c), but significantly (*P* < 0.05) decreased the expression of *dnmt3b* in testes (Fig. 4d). No effects were observed in 30-dpf juveniles after treatment at 25 µM from 0 to 6 dpf (Fig. 4e, f). However, the same dose administered between 20 and 30 dpf significantly (*P* < 0.05) increased *dnmt1* expression at 30 dpf (Fig. 4g), while *dnmt3b* expression was not affected (Fig. 4h).

### Effects of 5-aza-dC on the ovarian transcriptome of treated females include downregulation of reproduction-related signalling pathways and a repression of genes related to epigenetic regulatory mechanisms

**Fig. 5:**
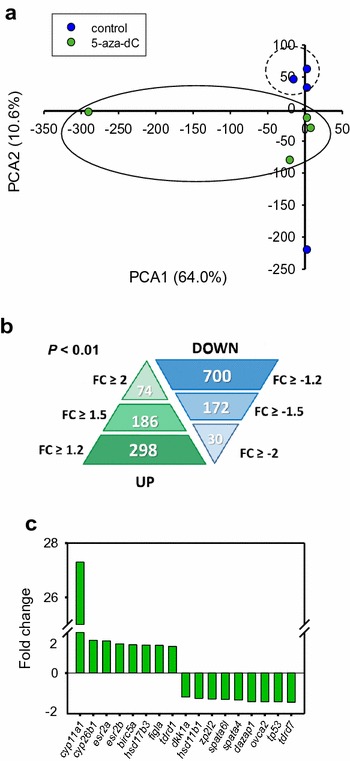
Transcriptomic effects in ovaries treated with 75 µM of 5-aza-dC during gonadal development (10–30 dpf). **a** Clustering of fish based on their ovarian transcriptomic profiles at 90 dpf as assessed by principal component analysis (PCA). Per cent values refer to variance (total variance explained ~ 75%). **b** Description of the number of differentially expressed genes (white numbers) according to different fold change values with a *P* < 0.01. **c** Gene expression at 90 dpf in zebrafish ovaries of some reproduction-related genes present in the microarray. Full gene names are listed in Additional file 5: Table S2

Since treatment with 5-aza-dC affected sex ratios by increasing the number of females, we wanted to examine the ovarian transcriptome of females resulting from exposure to 5-aza-dC. To do this, we compared females treated with 5-aza-dC at 75 µM from 10 to 30 dpf during the gonadal development period (Fig. 3c) with untreated females from the control group (*n* = 4 fish per group) as that was the group that showed the highest differences in growth in comparison with the lowest concentration (25 µm), suggestive of clear treatment effects. Expression profiles using a zebrafish homologous microarray (see materials and methods) were subjected to principal component analysis (PCA), which classified the samples into two clusters corresponding to control and treated fish. The PCA component 1 alone explained 64.0% of the variance, while component 2 explained an additional 10.6% (Fig. 5a). Between the two groups, there were a total of 998 differentially expressed genes (DEG), with 298 up- and 700 downregulated genes with a fold change (FC) ≥ 1.2 including both upregulation and downregulation and a *P* value < 0.01 (Fig. 5b). Likewise, the number of up- and downregulated DEG with a FC ≥ 2 was 74 and 30, respectively (Fig. 5b). Validation by quantitative (q) PCR using DEG between the two groups, and primarily related to reproduction, showed that the results obtained matched those obtained with the microarray (*R* ^2^ = 0.963, *P* < 0.0001), thus validating the microarray data (Additional file 2: Fig. S2).

In all gene ontology (GO) terms identified by analysing level 3, a larger number of downregulated GO terms were found (76) in comparison with the upregulated terms (43) (Additional file 3: Table S1, Additional file 4: Fig. S3). In the upregulated terms in the Biological process category (Additional file 4: Fig. S3A), the most enriched subcategories were: multicellular organism development (GO:0007275), system development (GO:0048731) and animal organ development (GO:0048513). The most downregulated subcategories (Additional file 4: Fig. S3D) were: cellular nitrogen compound metabolic process (GO:0034641), cellular macromolecule metabolic process (GO:0044260) and nucleobase-containing compound metabolic process (GO:0006139). We also found a downregulation of a GO term directly related to methylation: methylation-dependent chromatin silencing (GO:0006346) (Additional file 4: Fig. S3D). In the Cellular component category (Additional file 4: Fig. S3B, E) only seven GO terms were characterized in the upregulated subcategory. Likewise, we identified 20 downregulated subcategories, most of them with a high significant enriched *p* value (*p* = 1E^−23^–E^−4^), i.e. intracellular (GO:0005622) and intracellular part (GO:0044424). For the Molecular process (MP) category (Additional file 4: Fig S3 C, F) the most enriched downregulated GO term was related to epigenetics: chromatin binding (GO:0003682).

We identified a total of 24 DEG involved in reproduction-related functions [43, 45–47] (Additional file 5: Table S2) of which 17 are shown in Fig. 5c. The most upregulated gene (FC = 27.30) was cytochrome P450 family (cyp) 11, subfamily a, member 1 (*cyp11a1*), which is involved in the glucocorticoid and steroid pathways catalysing the conversion of cholesterol to pregnenolone during gonad formation [48]. We found other gene members of the cytochrome P450 superfamily also upregulated: 17β-hydroxysteroid dehydrogenase 3 (*hsd17b3*), which is predominantly expressed in the testis, catalysing the conversion of androstenedione to testosterone [49] and cytochrome P450 family 26 subfamily B member 1 (*cyp26b1*), which degrades retinoic acid [50]. In contrast, *hsd11b1*, which catalyses the conversion of the stress hormone cortisol to the inactive metabolite cortisone [51], was downregulated, together with other male-related genes such as spermatogenesis-associated (*spata*) 6 like *spata6* *l*, *spata4* and azoospermia-associated protein 1 (*dazap1*). The oestrogen receptor (esr) 2a, *esr2b* and folliculogenesis-specific BHLH transcription factor (*figl*) were upregulated, whereas zona pellucida glycoprotein 2 (*zp2l2*) and ovarian tumour suppressor candidate 2 (*ovca2*) genes were downregulated.

**Fig. 6:**
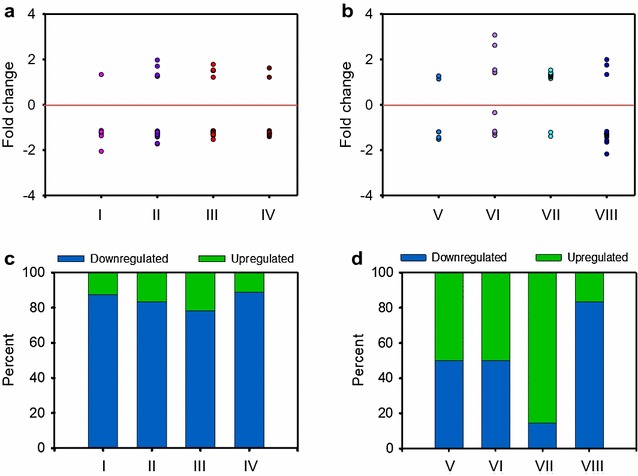
Lasting effects of treatment of zebrafish with 5-aza-dC at 75 µM between 10 and 30 dpf during gonadal development on the signalling pathways observed in 90-dpf adult ovaries. **a** Gene expression fold change differences in pro-female pathways. I: Fanconi anaemia (*n* = 8 genes), II: Wnt signalling (*n* = 30 genes), III: oocyte meiosis (*n* = 23 genes) and IV: progesterone-mediated oocyte maturation (*n* = 18 genes). **b** Gene expression fold change differences in pro-male pathways. V: PPAR signalling (*n* = 10 genes), VI: cardiac muscle contraction (*n* = 10 genes), VII: cytokine–cytokine interaction (*n* = 14 genes) and VIII: p53 signalling (*n* = 18 genes). **c**, **d** Percentage of up- or downregulated genes in the pro-female and pro-male pathways observed in **a** and **b**, respectively

We identified three enriched Kegg pathways that were upregulated: focal adhesion (dre04510), oxidative phosphorylation (dre00190) and regulation of actin cytoskeleton (dre04810) as well as ten downregulated Kegg pathways (Additional file 6: Table S3). Among them, the most enriched was the mTOR signalling pathway. We also found one reproduction-related pathway, progesterone-mediated oocyte maturation (dre04914), which was also inhibited. Next, we looked at four pathways typically associated with female development in zebrafish [43] (I: Fanconi anaemia, II: Wnt signalling, III: oocyte meiosis and IV: progesterone-mediated oocyte maturation), as well as four pathways typically associated with male development (V: PPAR signalling, VI: p53 signalling, VII: cytokine–cytokine interaction and VIII: cardiac muscle contraction) [43]. The fold change in the expression of genes belonging to these eight signalling pathways in the ovaries of 5-aza-dC-treated females compared to control females ranged approximately from − 2 to + 3 (Fig. 6a, b and Additional file 7: Table S4). Thus, in each pathway there were upregulated and downregulated genes. However, in the four pro-female pathways most genes were downregulated and these accounted for 78–90% of the total number of genes in each pathway (Fig. 6c). In contrast, the number of up- and downregulated genes was similar in two of the pro-male pathways (PPAR and p53 signalling), while in the cytokine–cytokine interaction pathway the number of upregulated genes clearly predominated, whereas in the cardiac muscle contraction pathway it was the opposite (Fig. 6b, d).

**Fig. 7:**
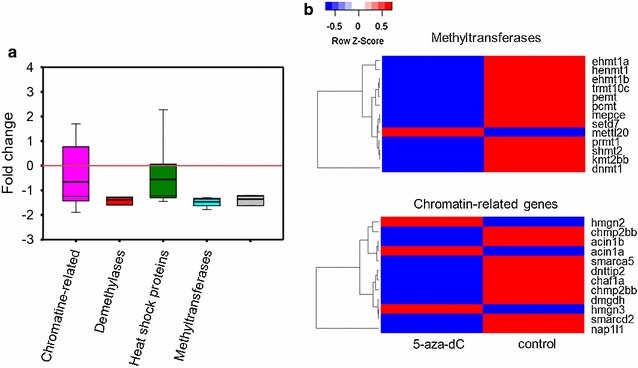
Expression of genes involved in different types of epigenetic regulatory mechanisms in the ovaries of 90-dpf adult zebrafish treated with 75 µM of 5-aza-dC during gonadal development (10–30 dpf). **a** Fold change with respect to control values set at 0. In the boxplot, the solid and thick lines indicate the median and mean, respectively; the lower and upper edges indicate the 25th and 75th percentiles, respectively; the lower and upper whiskers indicate the 5th and 95th percentiles, respectively. **b** Heatmap analysis of methyltransferases and chromatin-related genes in treated ovaries. Full gene names are listed in Additional file 8: Table S5

Finally, we investigated genes with a known epigenetic-related function and we found a total of 40 DEG which were classified according to their regulatory mechanisms [35, 52] (Fig. 7 and Additional file 8: Table S5): chromatin-related, e.g. chromatin assembly factor 1 subunit A (*chaf1a*); CpG binding domain, e.g. methyl–CpG binding domain protein 3a (*mbd3a*); demethylases, e.g. lysine (K)-specific demethylase 5Bb (*kdm5bb*); dicer, e.g. ribonuclease type III (*dicer1*); histone-related, e.g. histone cluster 1 H4c (*hist1h4c*), methyltransferases, e.g. calmodulin–lysine N–methyltransferase (*camkmt*), and polycomb-associated proteins, e.g. chromobox homologue 4 (*cbx4*). Regardless of their mechanism, 35 out of 40 of these genes were downregulated in the ovaries of treated females (Fig. 7a). These included all but one methyltransferase and all but three chromatin-related genes (Fig. 7b).

## Discussion

Changes in the methylome are part of normal development and occur throughout life in all vertebrates [53]. These changes can be artificially induced by DNA methylation inhibitors, the development of which has been fuelled for their promise in the treatment of some types of cancer. In fish such as medaka, *Oryzias latipes* [54], zebrafish [27] or goldfish, *Carasius auratus* [55], the effects of early treatment with DNA methylation inhibitors have been studied. Here we report the effects of 5-aza-dC treatment applied during either early development or gonadal development in zebrafish. We show for the first time that this DNA inhibitor is able to affect not only the process of sexual development but also the gonadal transcriptome of adults.

Regarding early developmental treatments, these were conducted within the first 2 h after fertilization, since a previous study in zebrafish already showed that such treatments indeed result in a global DNA hypomethylation [27]. In our study, early treatment with 5-aza-dC resulted in lower survival, which was evidenced eight days later. In fact, the toxicity of 5-aza-dC, first reported in human cells, prompted the development of less toxic and more stable agents such as zebularine [19, 56]. Cytotoxic effects have also been reported in medaka [28] and are thought to be due to induced DNA hypomethylation, emphasizing the importance of DNA methylation for proper development [57]. When the 5-aza-dC dose was 75 µM and exposure included most of the gonadal development (10–30 dpf) not only survival but also growth was reduced. This reduction in growth is in accordance with a previous study in which zebrafish was fed with 5-aza-dC at 10 mg/kg for 32 days and where adult females had reduced growth [32].

One of the most striking results found in this study is that 5-aza-dC treatment consistently resulted in a sex ratio bias towards females in all the experiments performed. This is in line with the results obtained with an hermaphroditic fish, the ricefield eel, *Monopterus albus*, in which 5-aza-dC treatment drove the natural sex reversal towards female development [58]. Our study shows that 5-aza-dC at 75 µM applied during 48 hpf was capable to feminize zebrafish. When the exposure time was longer (6 dpf) but the drug was administered in lower concentrations (15 and 25 µM) similar results were obtained. Further, when exposure lasted longer and included the gonadal development period (20–30 dpf) the number of females was also significantly increased. In contrast, in a recent study in which zebrafish was treated with 5-aza-dC during 0–6 dpf, no differences in sex ratios were found in the F0, but a shift towards males was observed in the F1 [33]. However, in that study group spawnings were used and it is thus difficult to ascertain the genetic contribution of parents, which can heavily influence the results [43, 46]. Our results are unlikely due to differential mortality because the early treatment experiments ended at 6 dpf at the latest, well before the differentiation of the gonads in zebrafish. Adult stickleback, *Gasterosteus aculeatus*, fed with 5-aza-dC (5 µg/g feed) had global DNA changes in both testes and ovaries, with significant changes in methylation levels particularly in the testes, indicating that 5-aza-dC is also able to affect differentiated gonads [31]. Interestingly, treatment with 5-aza-dC of children suffering from acute leukaemia resulted in hypogonadism, indicating that 5-aza-dC can affect the reproductive tissues in humans as well [59]. Taken together, our results with zebrafish along with the results in the other fish species mentioned above suggested that epigenetic mechanisms involving DNA methylation—for example, by decreasing methylation of the *cyp19a1a* promoter in ricefield eel [58]—suggest the possibility of testing DNA methylation inhibitors as a new option to control fish sex ratios as done, for example, in fish farming or population control. Nevertheless, further research should be carried out in additional species.

Transcriptomic data from the ovaries of treated zebrafish with 75 µM of 5-aza-dC during gonadal development showed changes in the expression of genes related to reproduction and sex differentiation. We identified three affected genes of the P450 cytochrome family. The *cyp11a1*, coding for a key enzyme implicated in steroid biosynthesis, was the highest upregulated reproduction-related gene in ovaries of treated zebrafish, although normally higher expression levels are found in testes [43]. This gene is mostly expressed in the brain, head, kidney and testis of adult fish, but it has also been involved in ovarian formation [48, 60]. In humans, the expression levels of *cyp11a1* also increased in placental cells treated with 5-aza-dC [61]. The *cyp261b* gene, which was also upregulated, is involved in the sexually dimorphic entry into meiosis in mammals (downregulation in ovaries and upregulation in testis) [62, 63]. In zebrafish this role seems to be carried on by *cyp261a* [45], although higher levels of *cyp261b* were found in testis of adult heat-treated males when compared to control female ovaries [43]. The *cyp17a1* gene was upregulated in 5-aza-dC-treated lymphocytes of infertile men [64], but it was downregulated in our study. We also identified an upregulation of two oestrogen receptors (*esr2a* and *esr2b*), similar to was observed in breast cancer cultured cells treated with 5-aza-dC [65]. In addition, we identified a downregulation of ovarian tumour suppressor candidate 2 (*ovca2*) gene similar to that occurred in 5-aza-dC-treated lung cancer cell lines [66].

Zebrafish is a gonochoristic species in which all individuals initially differentiate female-like gonads. Then, the gonads of about half of the fish enter apoptosis and are transformed into testis [67]. Zebrafish treated with 5-aza-dC showed an increase of the number of females; thus, the transformation into testis might have been interrupted due to the treatment. Treatment also resulted in an inhibition, persisting in adults, of four signalling pathways associated with female development, while one out of four pro-male pathways was clearly upregulated.

This is in agreement with the observation that some male-related genes and male-related pathways were found upregulated in ovaries of exposed fish, indicating that permanent but hidden effects of the treatment during sex differentiation lasted until adulthood. When exposing zebrafish to elevated temperature some females remain as such and others become masculinized. However, recently we discovered that some of the heat-resistant zebrafish females have a male transcriptome [43]. Thus, regardless of exposure, either to heat or to a demethylation agent, some zebrafish females exhibit resistant ovaries in terms of form, although they present a transcriptome similar to that of male gonads.

Epigenetic regulatory mechanisms are implicated in sex determination/differentiation in a wide variety of organisms, including plants and animals (reviewed in [35]). Thus, we also examined genes such demethylases, chromatin-related and histone-related genes. The expression of most of these genes was inhibited long after the end of treatment. This resembles the situation after cancer therapy, where demethylation agents are administered during 3–6 months [68, 69], but side effects such as hematologic and renal toxicities can persist 9–13 months later [70]. The effects of 5-aza-dC are thought to be limited to euchromatin, suggesting some sort of genome selectivity [71]. However, how this favours the development and maintenance of the female phenotype is not known.

The observed upregulation of some genes related to the epigenetic machinery (e.g. *dicer1*, *ehmt2*, *hdac11*, *mettl22*) is consistent with observations in European sea bass juveniles after early exposure to heat [52]. We also found a repression of 12 out of 13 methyltransferases (e.g. *mepce*, *kmt2ab*, *ehmt1a*, *prmt1* and *dnmt1*). These enzymes catalyse the transfer of methyl groups on histones [72]. Some methyltransferases have been identified in the gonads of fish, for example *prmt5*, which is implicated in oogenesis and spermatogenesis [73], and *ehmt2*, the transgenerational regulation of which was recently reported in the testis [74, 75]. Due to its important role in DNA methylation of the genome, dnmts are the most well-studied methyltransferases, not only in mammals, but also in fish. 5-Aza-dC treatment results in a hypomethylation of the genome because the complex DNA-5-aza-dC depletes the activity of the dnmts by the proteasome pathway and activates gene expression [18, 76, 77]. We found inhibition of *dnmt3b* in adult testes of fish treated for 48 h with 75 µM of 5-aza-dC, but gene expression of the two major *dnmts* in ovaries was not affected. However, when treatment included the gonadal development (10–30 dpf) period, a significant inhibition of *dnmt1* expression in the ovaries at 90 dpf was observed (Additional file 8: Table S5) despite that just at the end of the treatment it expression was increased (Fig. 4g). These results are in agreement with observations made in Japanese ricefield eel embryos, where *dnmt1* and *dnmt3b* expression was enhanced after 5-aza-CR treatment during 2 dpf [28]. *Dnmt3aa* and *dnmt3bb.1* were also upregulated in hatched embryos of *Solea senegalensis* after 24 h of 5-aza-CR treatment, while *dnmt1* was downregulated [78]; in zebrafish larvae treated with 5-aza-dC during 0–6 dpf, where *dnmt3bb.2* was upregulated, whereas *dnmt1* was not [33]. Thus, the responsiveness to 5-aza-dC treatment can be variable as this drug has multiple in vivo targets [79].

*Dicer1* is a key enzyme required for miRNA formation, and so this gene is involved in transcriptional repression functions [80]. Dicer1 is crucial for oocyte maturation in invertebrates [81], and its depletion renders sterile females in insects [82]. In zebrafish, *dicer1* has no role in oocyte maturation as primordial germ cells proliferate and remain pluripotent to form the adult germ line in the *dicer1* mutant [83]. However, *dicer1* is essential for zebrafish development [84]. In this study, we found *dicer1* downregulated in the ovaries after 5-aza-dC treatment during sex differentiation process together with other epigenetic-related genes. We also found downregulation of genes of the polycomb group, which are also repressors of the gene transcription machinery, in particular, the polycomb homologue 1 (*epc1*) and two chromobox genes (*cbx4* and *cbx5*). In mammals, the role of the *cbx2* in sex determination and differentiation has been shown [85, 86], whereas in Nile tilapia it was demonstrated that the expression of *cbx1b*, *cbx3a* and *cbx5* was sexually dimorphic in the gonads [87].

## Conclusions

We studied the effects of DNA methylation on vertebrate sexual development in a well-established model, the zebrafish. We report that the demethylating agent 5-aza-dC results in a sex ratio bias towards females in this species. The scarce data in other species point also to this direction, but whether this is a truly generalized effect is at present unknown. Thus, our results show the importance of DNA methylation for proper control of sexual development and open new avenues for the potential control of sex ratios in fish (aquaculture, population control). We also show that gene expression patterns of reproduction and epigenetic-related genes are affected by 5-aza-dC treatment in gonads, suggesting underlying DNA methylation changes that should be further studied. The long-term effects of treatment with 5-aza-dC at the time when the gonads are still not differentiated on the resulting adult gonadal transcriptome should be considered and explored in other situations. This could include, for example, prepuberal children treated with DNA-demethylating drugs as part of cancer therapy, given the fact that hypogonadism resulting from these treatments has already been reported.

## Methods

### Animal rearing conditions

Domesticated zebrafish (AB strain) were housed in 2.8-l plastic tanks (mod. ZT280) placed in a close-circuit system (Aquaneering, San Diego, CA, USA) inside a thermoregulated walk-in chamber at the aquarium facility of our institute. Inside the chamber the conditions were as follows: 12-h light/12-h dark constant photoperiod, air temperature of 26 ± 1 °C and humidity of 50 ± 3%. The water quality parameters were monitored daily (temperature: 28 ± 0.2 °C; pH: 7.2 ± 0.5; conductivity: 750–900 µS; dissolved oxygen: 6.5–7.0 mg l^−1^). Ammonium, nitrite, nitrate, silicate and phosphates were checked 2–3 times monthly by the water analysis service of our institute to ensure they remained in the appropriate ranges [41]. Fish were fed ad libitum three times a day with a commercial food (AquaSchwarz, Göttingen, Germany) according to their developmental stages and supplemented with live *Artemia* nauplii (AF48, INVE Aquaculture, Dendermonde, Belgium). Fertilization always followed natural spawning involving single-pair crossings. Batch size and fertilization rates were determined for each clutch to ensure values within accepted range for this species [41]. Likewise, it was ensured that post-hatch survival in the untreated groups was in accordance with the OECD’s guidelines for the Fish Sexual Development Test [88]. In order to avoid unwanted masculinization due to elevated rearing density, the number of fish per tank was kept in the range 25–35, based on our previous study of effects of density on zebrafish sex ratios [89].

### Ethics statement

Fish were kept in agreement with the European regulations of animal welfare (ETS N8 123, 01/01/91). Our fish facilities are approved for animal experimentation by the Ministry of Agriculture and Fisheries (certificate number 08039–46–A) in accordance with the Spanish law (R.D. 223 of March 1988). The experimental protocol was approved by the Spanish National Research Council (CSIC) Ethics Committee within the project AGL2013–41047–R.

### 5-Aza-dC treatments

#### Early development experiments

Fertilized eggs were reared at 26 ± 1 °C in 6-well Petri dishes (Thermo Fisher Scientific, Waltham, MA, USA) at 30 eggs/well filled with embryo medium (EM, pH 7.2 ± 0.5) supplemented with 0.1% methylene blue (Sigma-Aldrich, Madrid, Spain). Starting within the first 2 h post-fertilization (hpf), when zebrafish is sensitive to 5-aza-dC treatments [27], embryos were treated with 5-aza-dC (A3656, Sigma-Aldrich, Saint Louis, USA) added to the EM at a final concentration of 0 (control), 5, 15, 25 or 75 µM (Additional file 9: Fig. S4).

For the 5-, 15- and 25-µM concentrations, treatment lasted until 6 dpf. At 2 and 4 dpf, 50% of the EM volume was replaced with EM containing fresh 5-aza-dC at the appropriate concentration. At 6 dpf, fish were counted and thoroughly rinsed in EM and then housed in the 2.8-l tanks described above. At 30 dpf fish were counted again and a random sample (*n* = 7) of fish were killed and a cross section of the body trunk was cut and flash-frozen in liquid nitrogen until analysis. The remaining fish were left alive until 90 dpf. The experiment was replicated twice using eggs originating from the same breeding pair and involved a total of ~ 600 fertilized eggs.

For the 75-µM concentration, treatment lasted only from 0 to 2 dpf, since preliminary trials showed that at this concentration survival was unacceptably low with longer durations. At 1 dpf, 50% of the EM volume was replaced with EM containing fresh 5-aza-dC. At 2 dpf embryos were thoroughly rinsed in EM and reared in untreated EM. At 4 dpf, 5 pools of 20 larvae each from two technical replicates were collected from the 0- and 75-µM group. Larvae were flash-frozen in liquid nitrogen and kept at − 80 °C for further analysis. At 6 dpf, the remaining larvae were housed in the 2.8-l tanks described above. The experiment was repeated seven times using seven different breeding pairs, involving a total of ~ 1260 fertilized eggs.

The effects of all tested concentrations on survival, hatching rate and teratology were monitored daily until 8 dpf. At 90 dpf, fish were euthanized on iced water followed by severing the spinal cord. Survival, growth and sex ratios were recorded. To assess sex in adults, we used visual inspection of the gonad under a dissecting microscope as previously described [53]. Fish sex was determined individually after dissection, and the sex ratio was calculated for each biological replicate. Gonads were carefully dissected and flash-frozen in liquid nitrogen and stored at − 80 °C until further analysis (Additional file 9: Fig. S4).

#### Gonadal development experiments

This experiment targeted the period of gonadal sexual development in zebrafish [38, 40]. The effects of 5-aza-dC at 25 µM were studied in three different periods: 10–20, 20–30 and 10–30 dpf. In the latter period, the concentration of 75 µM was also tested (Additional file 9: Fig. S4). For each period, 2–4 technical replicates were used and the whole experiment was repeated twice. For these experiments, larvae obtained from pooled eggs of five different broodstock pairs were used. To carry out this experiment, 10-dpf larvae were randomly assigned to 2.8-l tanks. Treatments were carried out with static bath at different nominal 5-aza-dC concentrations (0, 25 or 75 µM). The tanks were placed inside a large thermoregulated tub to ensure constant temperature. Three times week during the treatment period, 50% of the water in each tank was replaced with water containing fresh 5-aza-dC at the appropriate concentration. Once all treatments with 5-aza-dC were finished at 30 dpf, the water of all tanks was replaced and fish were still maintained in the tanks inside the tub for an additional 2 weeks to ensure complete clearance of the drug before being returned to the commercial rack.

Survival was recorded periodically every 5–10 days during the course of this experiment. At 30 dpf 12 juvenile fish (whole body) per group were flash-frozen individually and kept at − 80 °C for further gene expression analysis. At 90 dpf all remaining fish were killed and sampled as described above.

### Gene expression analysis

Tissues were homogenized with 0.5 ml of TRIzol (Sigma), and total RNA was extracted with chloroform, precipitated with isopropanol and washed with 75% ethanol. Pellets were suspended in 25 µl DEPC–water and stored at − 80 °C. Total RNA concentration was determined by spectrometry (ND-1000 spectrophotometer, NanoDrop Technologies), and quality was checked on a 1% agarose/formaldehyde gel. RNA (200 ng) was treated with DNAse I, Amplification Grade (Thermo Fisher Scientific Inc., Wilmington, DE, USA H) and retrotranscribed to cDNA using SuperScript III RNase Transcriptase (Invitrogen, Spain) and Random hexamer (Invitrogen, Spain) following the manufacturer’s instructions. Quantitative PCR (qPCR) was carried out with the SYBR Green chemistry (Power SYBR Green PCR Master Mix; Applied Biosystems). All qPCRs were run in triplicate in optically clear 384-well plates. Cycling parameters were: 50 °C for 2 min, 95 °C for 10 min, followed by 40 cycles of 95 °C for 15 s and 60 °C for 1 min. Finally, a temperature-determining dissociation step was performed at 95 °C for 15 s, 60 °C for 15 s and 95 °C for 15 s at the end of the amplification phase. qPCR data were collected by SDS 2.3 and RQ Manager 1.2 software, and relative quantity (RQ) values were calculated by the 2∆∆Ct method [90, 91]. Specificity for each primer pair was also confirmed by dissociation step, primers efficiency curves and PCR product sequencing. Primer sequences used for gene expression study are shown in Additional file 10: Table S6.

### Microarray hybridization and analysis

For microarray analysis, RNA samples (*n* = 4 for control and *n* = 4 for 5-aza-dC groups) from ovaries at 90 dpf of fish subjected to 75 µM of 5-aza-dC during 10–30 dpf were used. RNA integrity was measured by a Bioanalyzer 2100 (RNA 6000 Nano LabChip kit Agilent, Spain). Samples with a RNA integrity number (RIN) > 8.5 were used for microarray hybridizations. Briefly, 50 ng of total RNA was labelled using the Low Input Quick Amp Labeling Kit, One-Color (Cy3; Agilent Technologies). Samples were hybridized individually in a 4 × 44 K Agilent platform (G2519F) at the Barcelona Biomedical Research Park (PRBB). cRNA was prepared for overnight hybridization with the corresponding buffers during 17 h at 65 °C and washed on the following day. Hybridized slides were scanned using an Agilent G2565B microarray scanner (Agilent Technologies, USA). Agilent software was used to avoid saturation, and feature extraction generated the raw data for further preprocessing. Statistical analyses were carried out with the statistical language R (2.13.1 version). Array normalization was implemented using the Quantile method in the Limma package in R (http://www.R-project.org/). *P* value < 0.01 threshold was applied to identify genes that showed statistically significant differences in gene expression from comparisons of interest. Microarray analysis software Multiple Experiment Viewer (MeV) version 4.8.1 was used to analyse microarray data and visualized samples by PCA. For the heatmaps statistical language R (3.3.2 version) was used with the gplot package. The log2 transformation of the fluorescence values was used for the statistical analysis. DAVID Bioinformatic Resources 6.8 and REVIGO software [92, 93] was used to analyse and study the enriched gene ontology (GO) terms in the DEG between groups. For GO terms analysis, a Fisher exact test (*P* < 0.05) false discovery rate (FDR) corrected for multiple testing was performed using all genes in our microarray as background and the DEG of each comparison as query. Microarray data were submitted to NCBI’s Gene Expression Omnibus (GEO) [94] and are accessible through GEO Series accession number GSE93367 (https://www.ncbi.nlm.nih.gov/geo/query/acc.cgi?acc=GSE93367).

Microarray was validated by quantifying the gene expression of 16 genes by qPCR (Additional file 2: Fig. S2D). The genes and the primers used are listed in Additional file 10: Table S6. The RNAs of the same individuals used for microarrays were retrotranscribed, and qPCR was performed as previously described. Genes were selected based on their importance to reproduction (Additional file 5: Table S2) and epigenetics (Additional file 8: Table S5) in fish sex determination and differentiation [35, 43, 45–47, 52].

### Statistical analysis

Data normality and the homoscedasticity of variances were checked with the Kolmogorov–Smirnov’s and Levene’s tests, respectively. One-way analysis of variance (ANOVA) was used to detect possible differences among groups in survival, BW and SL. Post hoc multiple comparisons were made with the Tukey’s test. The Student’s *t* test was used to detect differences in gene expression analysis by using 2∆Ct values [91]. For sex ratio analysis, Chi-squared test with Yate’s correction was used [95]. All data analyses were performed with Stat Graphics software (version 17). Data were expressed as mean ± s.e.m. In all tests, differences were accepted as significant when *P* < 0.05.

## Additional files

**Additional file 1: Fig. S1.** Effects of zebrafish treatment with 5-aza-dC at 75 µM during the period of gonadal development (10–30 dpf). (A) External differences between control and 5-aza-dC-treated adult zebrafish females at 90 dpf. Scale in cm. (B) Body weight and (C) standard length of adults at 90 dpf. Data shown as mean ± s.e.m. (*n* = 8 and 6 males, and 11 and 4 females in control and 5-aza-dC groups, respectively). Within each sex, significant differences (*P* < 0.05 for males and *P* < 0.01 for females) in growth were determined by the Student’s *t* test and are indicated by different letters.

**Additional file 2: Fig. S2.** Microarray analysis. (A) qPCR validation of microarray results using 16 genes. (Only 15 datapoints can be seen due to overlap.) See Additional file 6: Table S3 for further primer information.

**Additional file 3: Table S1.** List of enriched GO terms (level 3) found in the ovaries of fish treated with 75 µM of 5-aza-dC between 10 and 30 dpf.

**Additional file 4: Fig. S3.** Third level of gene ontology terms of differentially expressed genes found by microarray analysis of ovaries of fish subjected to 75 µm of 5-aza-dC between 10 and 30 dpf during gonadal development. (A, B, C) show the upregulated GO terms, (D, E, F) show the downregulated GO terms, (A, D) biological process, (B, E) cellular component and (C, F) molecular function.

**Additional file 5: Table S2.** List of reproduction-related genes differentially expressed (*P* < 0.01) obtained by microarray analysis in ovaries of zebrafish treated with 75 µM of 5-aza-dC between 10 and 30 dpf.

**Additional file 6: Table S3.** List of enriched Kegg pathways found in the ovaries of fish treated with 75 µM of 5-aza-dC between 10 and 30 dpf.

**Additional file 7: Table S4.** List of genes found differentially expressed in eight pathways associated with female or male development, in the ovaries of fish treated with 75 µM of 5-aza-dC between 10 and 30 dpf.

**Additional file 8: Table S5.** List of epigenetic-related genes differentially expressed (*P* < 0.01) obtained by microarray analysis in ovaries of zebrafish treated with 75 µM of 5-aza-dC between 10 and 30 dpf.

**Additional file 9: Fig. S4.** Experimental design to study the effects of 5-aza-dC treatment on zebrafish development and survival, growth, sex ratio and gene expression. (A) Experiments performed during the early stages of development, with treatments either from 0 to 2 or 0–6 days post-fertilization. (B) Experiments performed during the gonadal development period. In the different experiments, 2–7 biological replicates per treatment were used, with 2–3 technical replicates each.

**Additional file 10: Table S6.** Gene symbols, names, Refseq IDs and primer sequences for all genes used in qPCR (in alphabetical order) in this study.

## Abbreviations

5-aza-dC: 5-aza-2′-deoxycytidine
dnmt: DNA methyltransferase
dpf: days post-fertilization
FC: fold change
GO: gene ontology
PCA: principal component analysis
