# Supplementary figures and images for: Treatment with a DNA methyltransferase inhibitor feminizes zebrafish and induces long-term expression changes in the gonads
Source: Epigenetics Chromatin. 2017 Dec 8;10:59. doi: 10.1186/s13072-017-0168-7 (PMC5721477; doi:10.1186/s13072-017-0168-7)

**A**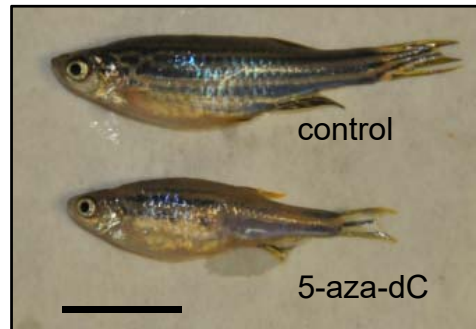**B**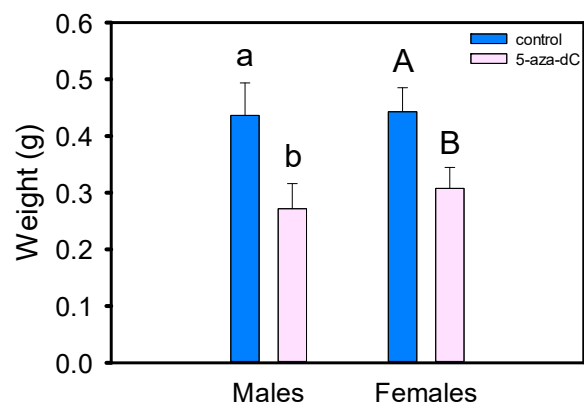**C**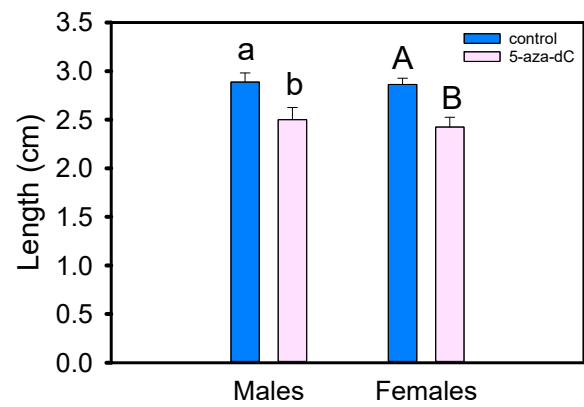

Supplement: Supplementary file 1 — Additional file 1: Fig. S1. Effects of zebrafish treatment with 5-aza-dC at 75 µM during the period of gonadal development (10–30 dpf). (A) External differences between control and 5-aza-dC-treated adult zebrafish females at 90 dpf. Scale in cm. (B) Body weight and (C) standard length of adults at 90 dpf. Data shown as mean ± s.e.m. (n = 8 and 6 males, and 11 and 4 females in control and 5-aza-dC groups, respectively). Within each sex, significant differences (P < 0.05 for males and P < 0.01 for females) in growth were determined by the Student’s t test and are indicated by different letters. [file 13072_2017_168_MOESM1_ESM.pdf]

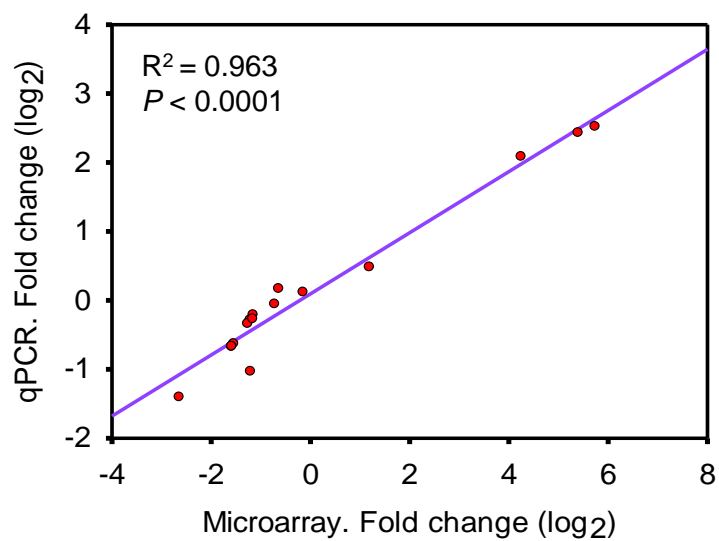

Supplement: Supplementary file 2 — Additional file 2: Fig. S2. Microarray analysis. (A) qPCR validation of microarray results using 16 genes. (Only 15 datapoints can be seen due to overlap.) See Additional file 6: Table S3 for further primer information. [file 13072_2017_168_MOESM2_ESM.pdf]

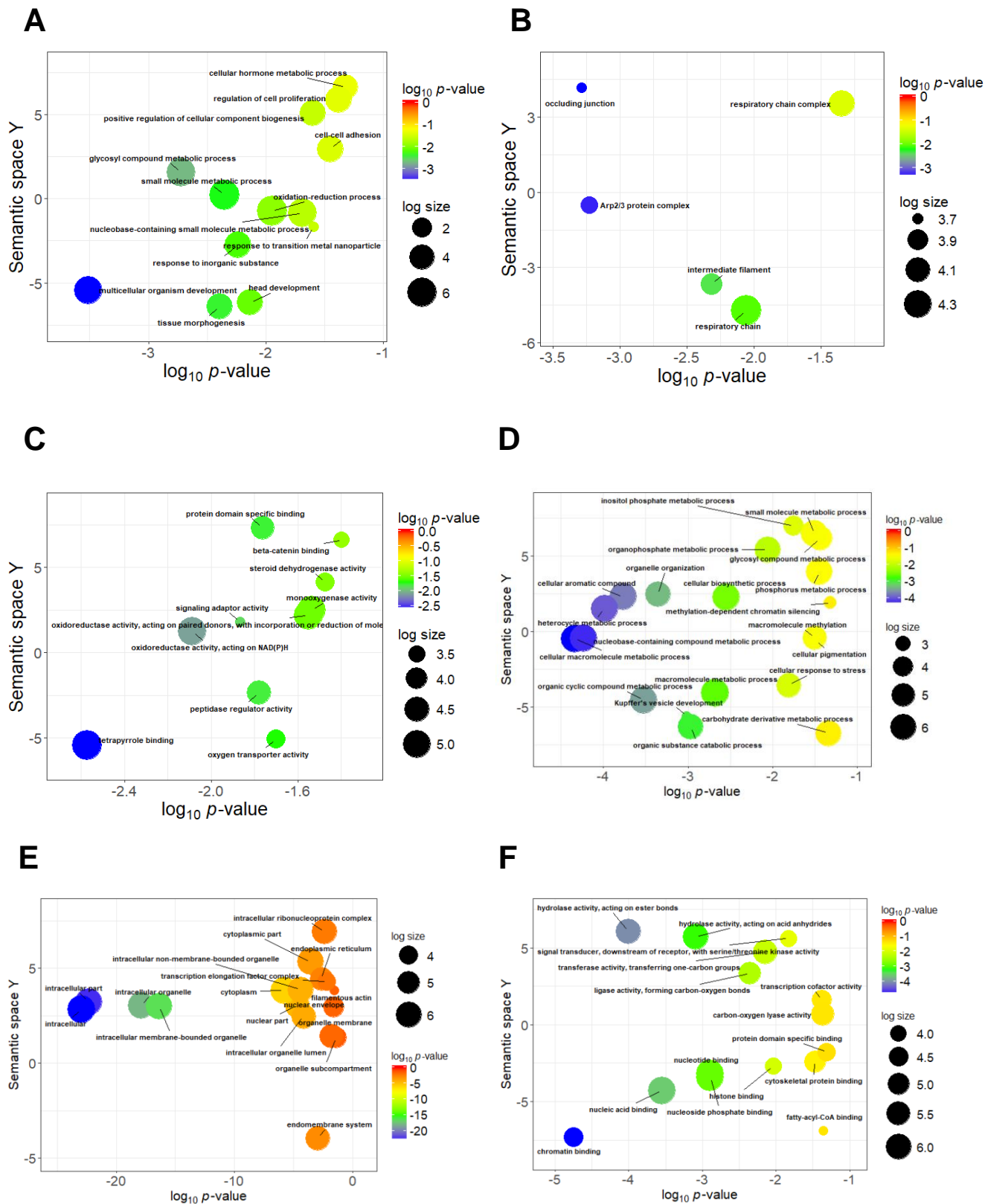

Supplement: Supplementary file 3 — Additional file 3: Table S1. List of enriched GO terms (level 3) found in the ovaries of fish treated with 75 µM of 5-aza-dC between 10 and 30 dpf. [file 13072_2017_168_MOESM3_ESM.pdf]

**A**

### Early stages of development

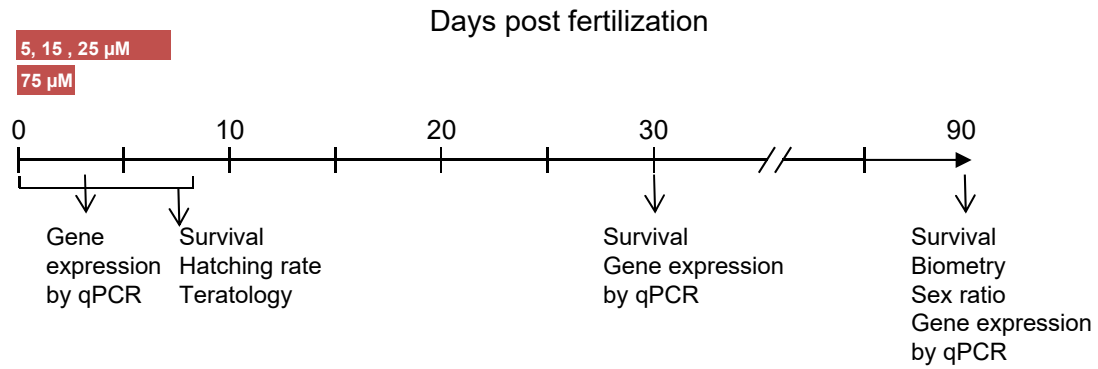**B**

### Gonadal development

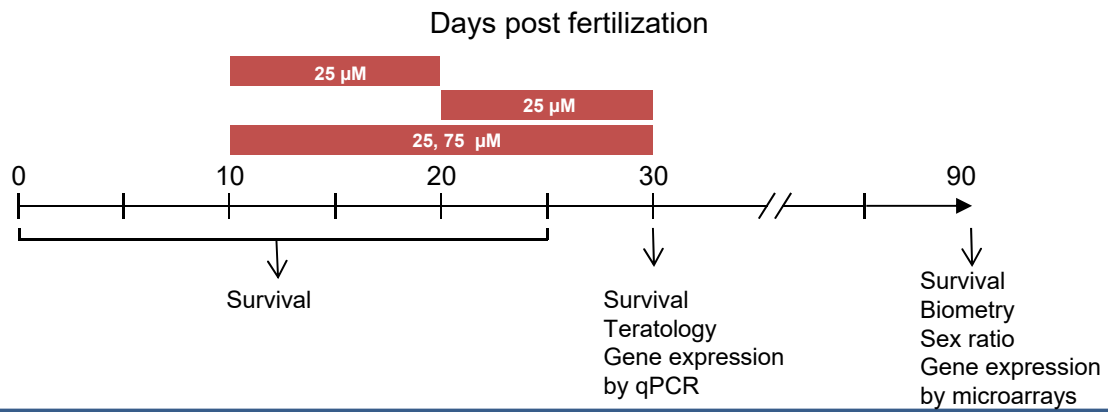

Supplement: Supplementary file 4 — Additional file 4: Fig. S3. Third level of gene ontology terms of differentially expressed genes found by microarray analysis of ovaries of fish subjected to 75 µm of 5-aza-dC between 10 and 30 dpf during gonadal development. (A, B, C) show the upregulated GO terms, (D, E, F) show the downregulated GO terms, (A, D) biological process, (B, E) cellular component and (C, F) molecular function. [file 13072_2017_168_MOESM4_ESM.pdf]
